# Water Recovery from Bioreactor Mixed Liquors Using Forward Osmosis with Polyelectrolyte Draw Solutions

**DOI:** 10.3390/membranes12010061

**Published:** 2021-12-31

**Authors:** Calen R. Raulerson, Sudeep C. Popat, Scott M. Husson

**Affiliations:** 1 Department of Chemical and Biomolecular Engineering, Clemson University, 127 Earle Hall, Clemson, SC 29634, USA; crauler@clemson.edu; 2 Department of Environmental Engineering and Earth Sciences, Clemson University, 342 Computer Court, Anderson, SC 29625, USA; spopat@clemson.edu

**Keywords:** anaerobic membrane bioreactor, forward osmosis, polyelectrolyte draw solution, reverse solute flux, water regeneration

## Abstract

This paper reports on the use of forward osmosis (FO) with polyelectrolyte draw solutions to recover water from bioreactor mixed liquors. The work was motivated by the need for new regenerative water purification technologies to enable long-duration space missions. Osmotic membrane bioreactors may be an option for water and nutrient recovery in space if they can attain high water flux and reverse solute flux selectivity (RSFS), which quantifies the mass of permeated water per mass of draw solute that has diffused from the draw solution into a bioreactor. Water flux was measured in a direct flow system using wastewater from a municipal wastewater treatment plant and draw solutions prepared with two polyelectrolytes at different concentrations. The direct flow tests displayed a high initial flux (>10 L/m^2^/h) that decreased rapidly as solids accumulated on the feed side of the membrane. A test with deionized water as the feed revealed a small mass of polyelectrolyte crossover from the draw solution to the feed, yielding an RSFS of 80. Crossflow filtration experiments demonstrated that steady state flux above 2 L/m^2^·h could be maintained for 70 h following an initial flux decline due to the formation of a foulant cake layer. This study established that FO could be feasible for regenerative water purification from bioreactors. By utilizing a polyelectrolyte draw solute with high RSFS, we expect to overcome the need for draw solute replenishment. This would be a major step towards sustainable operation in long-duration space missions.

## 1. Introduction

Treating crew waste streams for long-duration (i.e., 30 months) space missions will require regenerative approaches to maximize water (and nutrient) recovery and shift focus from waste removal to resource recovery [1]. Current Environmental Control and Life Support Systems (ECLSS) aboard the International Space Station (ISS) and other spacecraft are inadequate for addressing long-duration mission constraints [1]. Fecal and food wastes currently are not recycled [2], resulting in losses of valuable nutrients and water. Future ECLSS designs should take in all waste streams and process them with >98% water recovery—NASA’s goal for human missions to Mars—which would virtually eliminate the need to supplement the original supply [3].

Designs based on municipal wastewater treatments plants (WWTPs) are not feasible for ECLSS. Such facilities have large footprints encompassing many different processes designed to handle large intake volumes and complex feed compositions. WWTPs based on conventional activated sludge treatment also include primary clarifiers, grit removers/filters, aeration tanks, digesters, and additional clarification steps. Furthermore, they require addition of chemicals to treat the wastewater. High energy input is needed to aerate the bioreactors in the activated sludge, as the microorganisms need oxygen as an electron acceptor to break down organics. Large volumes of sludge are produced that need to be further processed. The resulting biosolids produced from activated sludge solids, once dewatered, may be converted into useful components such as fertilizer, biogas, and other fatty acids after digestion.

Instead, there is a significant opportunity to develop membrane bioreactors (MBRs) for incorporation in ECLSS for future long-duration space missions. These tend to require lower energy and chemical inputs, have high potential for water recovery, and operate under ambient conditions. These could especially be operated under anaerobic conditions, to yield additional advantages of energy recovery as biogas. Conventional anaerobic MBRs (AnMBRs) decouple hydraulic retention time (HRT) from solids retention time (SRT) by utilizing microfiltration or ultrafiltration membranes. This enables a long SRT that improves effluent quality, reduces waste biosolids production, and increases methane production [4]. However, low molecular weight organic species can pass through the membranes. Another pitfall results from the necessary applied pressure for filtration, leading to higher rates of fouling.

Osmotic membrane bioreactors (OMBRs) have a key advantage over standard MBRs. They rely on osmotic pressure differences to drive water transport across a forward osmosis (FO) membrane, resulting in lower rates of fouling [5,6]. Unlike microfiltration or ultrafiltration membranes, FO membranes have high rejection of low molecular weight organic species prolonging their retention time and facilitating biodegradation [4]. State-of-the-art thin-film composite (TFC) FO membranes provide high flux, low reverse salt transport, and good resistance to fouling. Further improvements in osmotic flux can be gained by replacing conventional non-woven backings with a more open woven mesh backing that decreases the effects of internal concentration polarization, as discovered by Idarraga-Mora et al. [7]. 

While FO solves some of the problems associated with traditional MBR operations, several problems persist, especially for applications in anaerobic OMBRs (AnOMBRs). The high rejection and buildup of phosphate and ammonia-nitrogen constituents is common and detrimental [8,9,10]. Like concentration polarization, this buildup reduces the osmotic driving force, leads to unsteady state operation, and may affect microbial activity. Another problem is the accumulation of the draw solute in the bioreactor. Historically, simple salts such as NaCl and MgCl_2_ have been used as draw solutes. During operation, there is inherent crossover of the draw solute into the bioreactor, so called reverse solute flux (RSF). Over time, the draw solute will need to be replenished, which should be avoided for long-term space missions. In addition, biological performance can be affected severely by salinity buildup and RSF. Chang et al. [11] reported declining flux over long periods of operation due to RSF and draw solution dilution when using MgCl_2_ as a draw solute. Flux decreased from 3.7 to 3.3 L/m^2^/h over 85 days and high salinity buildup was observed in the bioreactor.

A number of draw solutes have been evaluated for AnOMBR in terms of their osmotic pressure, water flux, RSF, and anaerobic treatment of draw solute-impacted substrate [12]. One measure of their effectiveness is the reverse solute flux selectivity (RSFS), a dimensionless number defined as the quotient of the mass of permeated water and the mass of draw solute that has diffused from the draw solution. A high RSFS is desirable for sustainable operation. Polyelectrolyte-based draw solutions have been used to combat the problems of draw solute accumulation in the feed and low RSFS experienced with conventional draw solutes. Polyelectrolyte solutions can achieve a high-water flux and low RSF due to their large size. Ge et al. [13] studied the roles played by polyelectrolyte solution concentration, viscosity, molecular weight, and temperature on water recovery from dye wastewater. They found that high concentrations and temperatures increased FO flux, while higher viscosities decreased flux. Significant flux decline arose from dilution of the draw solution. A continuous wastewater treatment process was established by integrated FO with membrane distillation (MD) [5]. MD continuously recovered water from the draw solution, thereby maintaining its concentration at a constant level. 

The objective of this study was to explore FO using polyelectrolyte draw solutions for regenerative water purification from bioreactor mixed liquor. Sodium salts of poly(acrylic acid) (PAA-Na) and poly(acrylic acid-*co*-maleic acid) (PAMA-Na) were evaluated as draw solutions with different specific charge densities (14.1 meq/g for PAA-Na and 16.0 meq/g for PAMA-Na). Wastewater from a municipal wastewater treatment facility and effluent from an anaerobic bioreactor were used to simulate wastewater obtained from the ECLSS. Direct flow and crossflow filtration experiments were performed to measure permeate flux and RSFS, assess membrane fouling, and establish feasibility for pseudo steady state operation over a 70-h test run. Findings from this work begin to address key challenges for implementing OMBRs in ECLSS for space applications.

## 2. Materials and Methods

### 2.1. Chemicals and Materials

PAA-Na (*M_w_* 2100) [9003-04-7] and PAMA (*M_w_* 3000) [29132-58-9] were obtained from MilliporeSigma (Burlington, MA, USA). PAMA was neutralized with sodium hydroxide (98% purity) sourced from Alfa Aesar (Haverhill, MA, USA) to form PAMA-Na. Acros Organics anhydrous ethanol (200 Proof, 99.5+%) was purchased from Thermo Fisher Scientific (Waltham, MA, USA). Type 2 deionized (DI) water (18 MΩ·cm) was produced with a Milli-Q water purification device (EMD Millipore, Billerica, MA, USA). 

Digested sludge obtained from an anaerobic methanogenic bacteria culture and primary effluent were kindly provided by ReWa (Greenville, SC, USA). ReWA provided data for average chemical oxygen demand (COD), total solids (TS), and volatile solids (VS) in the digested sludge over a 3-month period of operation. Average values were 26,494 ± 2714 mg COD/L, 2.08 ± 0.14 %TS, 1.47 ± 0.13 %VS. No data were provided for the primary effluent. The two fluids were mixed at a ratio of 20% digested sludge and 80% primary effluent (*v/v*) in a 15 L container. The fluids were mixed at the start of experimentation for each trial. No pretreatment was used prior to filtration. While our long-term vision is that AnOMBR will be the most effective approach, the current study was agnostic to the type of MBR. For convenience of operation, we performed all experiments under aerobic conditions. The feed was aerated using aeration stones for crossflow filtration tests, while the direct flow system was open to the air. Sulfur concentration in the effluent was representative of bioreactor digestors treating human waste ranging from 0.2 to 2.3 wt% [14].

Membranes (SW30HRLE) were provided by Dow Water & Process Solutions (Edina, MN, USA). Spacers with 1.8 ± 0.1 mm opening size and 440 ± 10 µm thickness were purchased from Sterlitech Corporation (Kent, WA, USA). Four spacers were utilized in crossflow experiments. 

### 2.2. Direct Flow Filtration Measurements

Direct flow filtration tests were conducted in a homebuilt apparatus resembling an osmotic pressure testing device. An illustration of the apparatus is provided in Supporting Information (Figure S1). The materials of construction were obtained from Grainger (Lake Forest, IL, USA). SW30HRLE membranes were wetted directly before use by soaking in a 50:50 (*v/v*) water/ethanol solution for 5 min and then placed in DI water for 10 min. A wetted membrane was placed between two rubber gaskets with the active layer facing the draw solution. The membrane and gasket assembly were sealed against the apparatus flanges with nuts and bolts. The flange joint was sealed tightly to prohibit leakage. The active area of the membrane was measured to be 472 mm^2^.

PAA-Na and PAMA-Na draw solutions were prepared with concentrations of 0.3 g/mL and 0.5 g/mL. Solid PAA-Na was dissolved in DI to produce 150 mL of draw solution. PAMA-Na was prepared by neutralizing PAMA with a stoichiometric amount of sodium hydroxide and adding DI water to produce 150 mL of draw solution. The feed consisted of 20:80 (*v/v*) digestor sludge/PCE. The conductivity of the feed was 1.4–2.1 mS/cm, within the typical range of bioreactor effluent. The solids concentration was 2.7–3.2 g/L, within the range of concentration from 2.2 g/L to 9.1 g/L for typical wastewater treatment facilities [15,16]. Stir bars with a length of 1 cm and a diameter of 0.8 cm were added to the feed and draw solution chambers to provide mixing at 1100–1400 rpm. The feed and draw solutions were added simultaneously and the apparatus was tilted to remove air bubbles that may have formed during pouring. Conductivity probes were inserted to each chamber to monitor concentration changes associated with water transport. Conductivity values were recorded by LabView 20 (National Instruments, Austin, TX, USA). Feed and draw solution height changes were recorded for flux calculations. These experiments were run for 8 h. 

RSFS was measured with the same apparatus by utilizing DI water as the feed solution and measuring conductivity over a period of 50 h to determine if any of the polyelectrolyte draw solute migrated across the membrane.

### 2.3. Crossflow Filtration Measurements 

Crossflow experiments were tested using the apparatus described in Idarraga-Mora [7] with modifications. A schematic of the modified apparatus is provided in Supporting Information, Figure S2. The apparatus was adapted from pressure-retarded osmosis operation to FO operation by changing the feed and draw solutions sides.

Membranes were wetted as described above and then installed in a custom membrane cell comprising two blocks of Delrin cut to form a crossflow channel with dimensions 44 mm (L) × 14 mm (W) × 2.35 mm (D). The membrane active area was 616 mm^2^. Four spacers were added on the draw solution side, following a previously described protocol [7]. The spacers gave structural stability to the membrane. The starting pressure ranged from 3.45 to 4.83 bar and was decreased to the operating pressure of 0.34–2.41 bar. 

LabView 2020 was utilized to automate operation of the system and record all data generated. Data collected included pressure, conductivity, flux, and pump speed. The feed solution comprising 20:80 (*v/v*) digested sludge/primary effluent was recirculated by a Hydra-Cell P100 Metering Pump (Wanner Engineering Minneapolis, MN USA) through 316 stainless steel tubing. Solutions of 0.3 g/mL PAA-Na and 0.3 g/mL PAMA-Na were recirculated using a MasterFlex L/S 600 rpm drive with an L/S Easy-Load II SS pump head (Cole-Parmer, Vernon Hills, IL, USA) through a combination of MasterFlex pump tubing (Puri-Flex, Cole-Parmer) and 316 stainless steel tubing. The flow rates for the feed and draw solutions were set to 0.5–0.7 L/min to maintain a crossflow velocity of 0.25 m/s. Conductivity probes (CS150TC, Sensorex Corporation, Garden Grove, CA, USA) were used to measure the changes in conductivity of feed and draw solutions. Draw solution mass was recorded continuously using an Ohaus AV3102C Adventurer Pro scale (Ohaus Corporation, Parsippany, NJ, USA) and used to calculate permeate flux in LabView. The pressure on each side of the membrane was measured. Overall and transmembrane pressures were kept as low as possible while maintaining the desired crossflow velocity. 

Time, conductivity, mass, and pressure measurements were recorded every minute over the 70 h experimental runs by the LabView software. Permeate flux was calculated from the mass change in the draw solution over time. Control of the pressure and flow rate of the feed solution was achieved by actuation of a MCJ050AB-3-SS-31RS4 proportional valve (Hanbay Laboratory Automation, Quebec, QC, Canada). The conductivity of the PAA-Na and PAMA-Na draw solutions were measured to be 46.6 mS/cm and 36.7 mS/cm at the start of each experiment.

## 3. Results

### 3.1. Direct Flow Measurements

**Figure 1:**
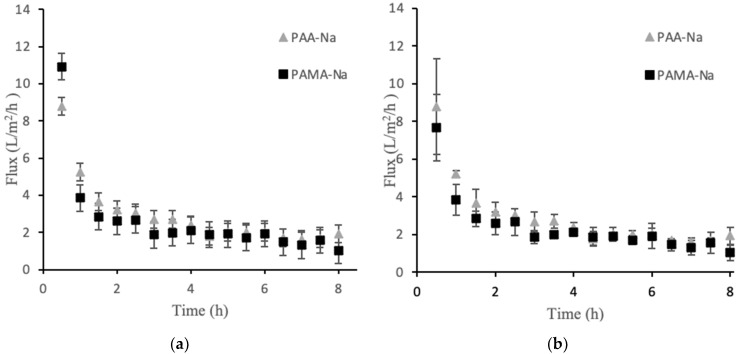
Direct flow flux decline data for PAA-Na and PAMA-Na at concentrations of 0.3 g/mL (**a**) and 0.5 g/mL (**b**). Error bars represent ± 1σ generated from three experiments.

Direct-flow measurements quantified the flux obtainable from the polyelectrolyte draw solutions. Figure 1a presents the permeate flux data for PAA-Na and PAMA-Na at 3 g/mL. There was an initial high flux value within the first 30 min that declined sharply over time. Figure 1b shows flux decline data for the higher draw solution concentration of 0.5 g/mL. The results were highly similar to experiments that used the lower concentration. The flux values after 1 h were the same within the experimental uncertainties. 

**Figure 2:**
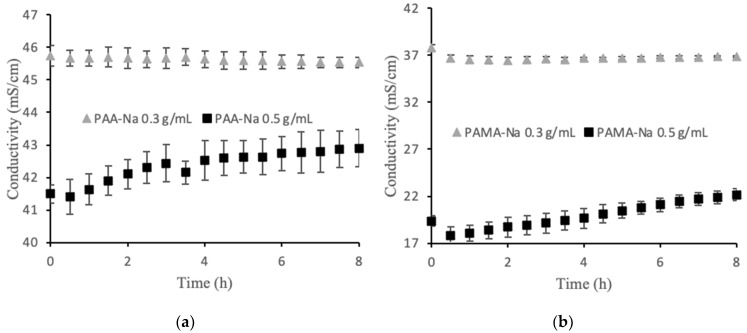
Direct flow conductivity values for (**a**) PAA-Na and (**b**) PAMA-Na. The error bars show one standard deviation from three separate experiments.

Among the factors that may contribute to flux decline are membrane fouling, decreased osmotic pressure driving force; external concentration polarization; internal concentration polarization; inadequate stirring; and changes in temperature, viscosity, and pH. For these experiments the temperature, viscosity, and pH were constant. A high stir speed was used on both sides of the membrane to minimize the effects of concentration polarization. The osmotic pressure driving force is expected to decline over time as the feed becomes concentrated and the draw solution is diluted by water permeation. Figure 2a,b show the conductivity values for two polyelectrolyte draw solutions. The 0.3 g/mL draw solutions showed no statistically significant change in conductivity over the 8 h runs. Thus, decreasing osmotic pressure driving force was not a significant factor for the observed flux decline in that case. An unusual observation was made for the 0.5 g/mL draw solutions in which conductivity steadily increased upon dilution. This observation is discussed in Section 3.2, but the result is that osmotic pressure driving force increased, which cannot explain the flux decline. Membrane fouling was the largest factor contributing to the flux decline, which explains the similarity in flux decline curves for the two draw solution concentrations. Solid particles accumulated on the feed side of the membrane forming a cake layer that increased in thickness over time. Post filtration, it was discovered that this layer was easy to remove mechanically, but visible observation indicated that some foulant remained within the porous layer. 

RSFS is another important metric to assess AnOMBRs for water and nutrient recovery in space, with values above 20 being considered feasible. During the RSFS test with DI water feed, the feed conductivity increased by 14.6 µS/cm in 51 h, resulting in a high RSFS value of 80.

### 3.2. Optimization of Polymer Concentration

**Figure 3:**
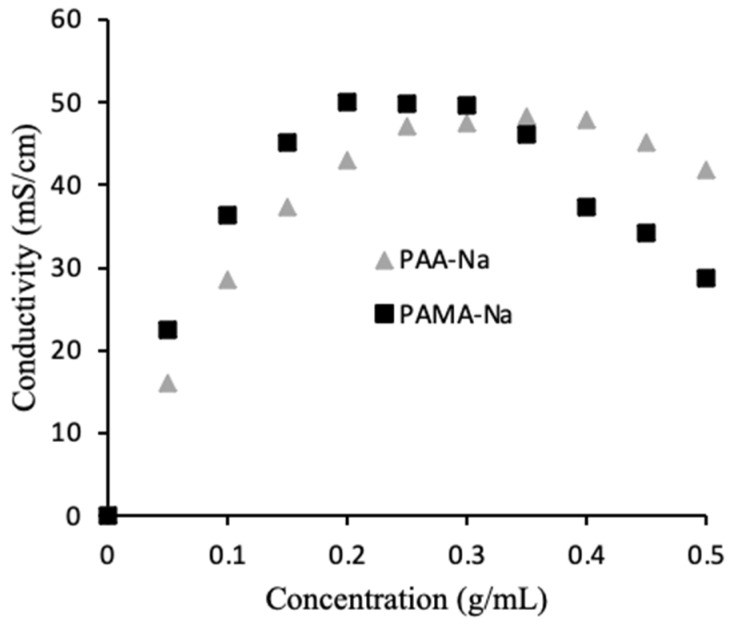
Effect of polymer concentration on draw solution conductivity.

As mentioned earlier, the conductivity of the 0.5 g/mL draw solutions unexpectedly increased upon dilution. To better understand this phenomenon, calibration curves were prepared to correlate conductivity to polyelectrolyte concentration. Figure 3 shows the results. For PAA-Na, the highest conductivity was measured at 0.35 g/mL, while the highest conductivity for PAMA-Na was measured at 0.20 g/mL. During the direct flow tests a similar trend was observed. Figure 2a shows increasing conductivity for the 0.5 g/mL PAA-Na draw solution upon dilution, whereas the conductivity of the 0.3 g/mL draw solution decreased slightly, as expected from results in Figure 3. The conductivity of the PAMA-Na draw solution increased with dilution for the 0.5 g/mL concentration and remained constant for 0.3 g/mL. The negligible conductivity changes for PAA-Na and PAMA-Na at 0.3 g/mL correspond with the plateau regions of Figure 3. These results also correspond with work done by Bordi et al. [17] showing that increasing concentration led to an increase in viscosity and a decrease in conductivity. They theorized that the higher concentration solutions demonstrated significant overlap of the polymer chains. Overlap of the polymer chains led to increased electrostatic interactions, excluded volume, hydrodynamic interactions, and pH changes resulting in a lowering of the conductivity.

**Figure 4:**
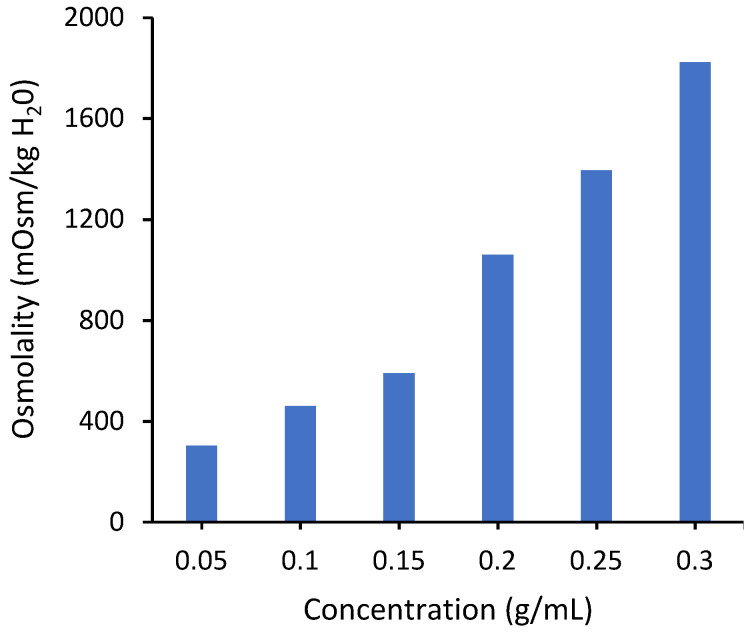
Osmotic pressure of PAA-Na measured by osmometry with the OsmoPRO Multi-Sample Micro-Osmometer from Advanced Instruments. The instrument was unable to determine the osmolality at concentrations higher than 0.3 g/mL due to a higher viscosity.

The osmotic pressure driving force depends on the conductivity of the draw solution. Figure 4 shows measured values of osmolality for PAA-Na solutions. The osmolality increases monotonically over the measured concentration range, consistent with the conductivity measurements. Unfortunately, it was not possible to measure osmolality for concentrations higher than 0.3 g/mL due to instrument limitations associated with the high solution viscosity. However, modeling based on the Flory–Huggins theory suggests that it will reach a maximum around 0.3 g/mL (Figure S9 in Supporting Information), similar to the conductivity curve in Figure 3, due to significant overlap of neighboring chains.

As the conductivity of the solutions is directly linked to the osmotic pressure, the draw solution concentrations will need to be optimized to increase permeate flux once fouling is managed. For long-term operation, a concentration slightly above that which produces the maximum in conductivity could be advantageous as the flux will remain nearly constant as the solution is diluted. In a continuous operation system, an additional RO or MD process could be employed on the draw side to recover pure water and reconcentrate the draw solution. Operating near the overlap concentration also will ensure that solution viscosity is not too high. High viscosity leads to higher energy input for pumping and exacerbates the CP and ICP. 

### 3.3. Crossflow Measurements

Crossflow filtration measurements were performed to test feasibility for longer term operation. Crossflow reduces the degree of fouling and decreases ICP and CP. During the crossflow experimentation the pressure was adjusted to maintain 0.25 m/s crossflow velocity. 

**Figure 5:**
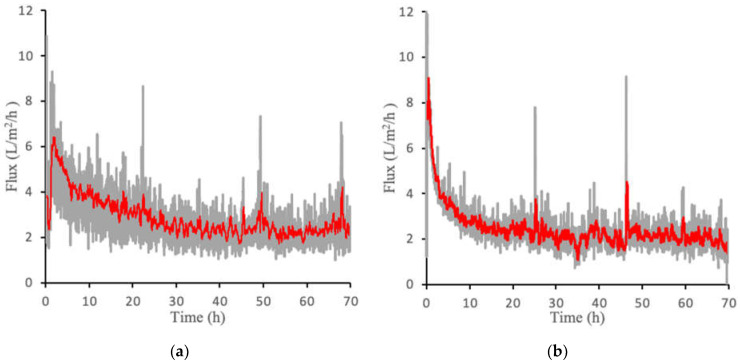
Crossflow flux measurements for (**a**) PAA-Na at 0.3 g/mL and (**b**) PAMA-Na at 0.3 g/mL. The gray lines represent the raw data generated from three separate experiments and the red line represents the 20-point moving average.

Figure 5a,b show the flux over a 70-h period for both PAMA-Na and PAA-Na at a concentration of 0.3 g/mL. Gray lines represent the absolute value of the flux, whereas the red lines display the 20-point moving average. The median percent error of the moving point average was determined to be 13% for PAA-Na and 11% for PAMA-Na. Similar to the direct flow results, the flux is highest at the beginning of the experiment due to the undeveloped fouling layer, a higher starting pressure, lower CP and ICP, and the highest osmotic pressure driving force exerted by the draw solution. Crossflow velocity was not optimized in these filtration experiments; thus, there is an opportunity to control fouling and concentration polarization during continuous operation by varying the crossflow velocity. In addition, it may be possible to use this system downstream of an ultrafiltration membrane or to use membrane sparging with biogas produced by the bioreactor to reduce fouling. Further improvements may come from application of membrane patterning [18] or replacing the nonwoven backing with a woven mesh as featured in Idarraga-Mora [7], which has been shown to be more effective for osmotic processes. PAMA-Na produced a slightly higher initial flux than PAA-Na. However, they both declined to roughly 2 L/m^2^/h at 30 h of operation and maintained this flux for the duration of the test. Initial experiments conducted for 143 h yielded the same results (Figure S8). For this reason, 70 h was deemed sufficient to establish these pseudo steady state flux profiles. 

**Figure 6:**
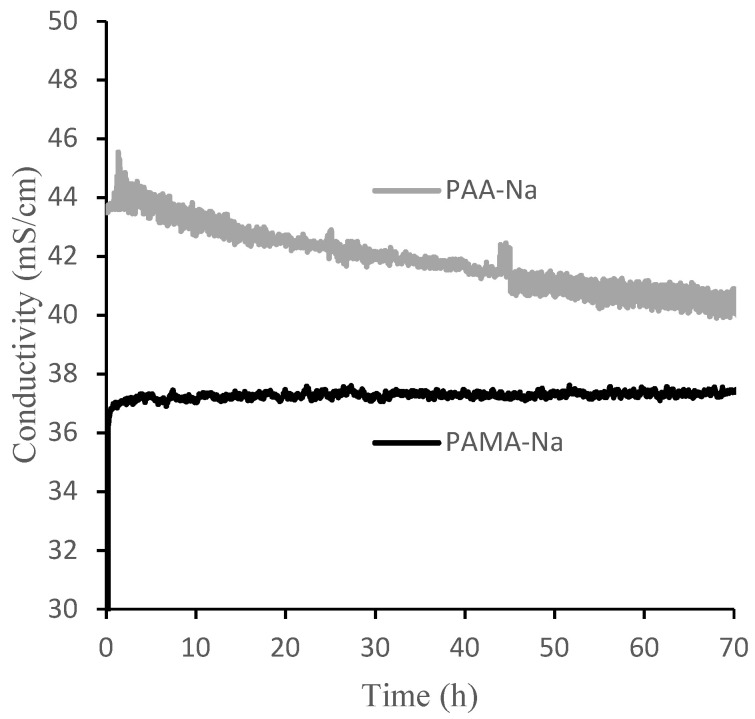
Conductivities of 0.3 g/mL PAA-Na and PAMA-Na draw solutions during 70 h crossflow filtration experiments.

Figure 6 shows measured conductivities of the draw and feed solutions over the 70-h experimental runs using 0.3 g/mL PAA-Na and PAMA-Na. Conductivity decreased monotonically for PAA-Na, resulting from dilution and a high rejection of the salts in the feed. Conductivity remained nearly constant for PAMA-Na. These trends can be explained by the calibration data in Figure 6, which show that PAA-Na conductivity decreases with decreasing concentration from 0.3 g/mL and the PAMA-Na conductivity remains constant with decreasing concentration from 0.3 to 0.2 g/mL. As the conductivity relates to the osmotic pressure driving force, draw solution concentrations should be selected to optimize the flux of the system. The feed solution conductivity increased slightly by 0.2–0.4 mS/cm throughout the 70-h test period corresponding to a high RSFS like that observed in the direct-flow filtration studies. 

## 4. Conclusions

Water recovery from bioreactor digester mixed liquors was achieved with adequate flux over long periods of time using forward osmosis with polyelectrolyte draw solutions. Fouling was the largest contributor to flux decline in direct flow measurements. A high initial flux quickly decreased as foulants accumulated on the porous side of the membrane, forming a cake layer that provided additional resistance to flow. A maximum in conductivity with polyelectrolyte concentration resulted in an optimum concentration for maximizing the osmotic driving force. As conductivity correlates directly to osmotic pressure, the draw solution concentration should be set slightly above the value corresponding to the maximum in conductivity to ensure that dilution does not lead to a substantial decrease in permeate flux. In a continuous operation system, an additional membrane distillation process could be employed on the draw side to recover pure water and reconcentrate the draw solution. Crossflow experiments demonstrated pseudo steady state operation and established the feasibility of using polyelectrolyte draw solutions for water extraction from human waste bioreactors, which is an important step towards developing a comprehensive understanding of the AnOMBR system for use in ECLSS and long-term water recovery systems.

Our results suggest that FO using polyelectrolyte draw solutions may be an option for space applications. The constraints on energy and atom efficiency in space make it a viable alternative to other treatment options like reverse osmosis. Looking forward, membrane fouling and concentration polarization will need to be managed to maintain a high osmotic pressure driving force. Membrane fouling could be managed using pretreatment by ultrafiltration, while concentration polarization could be managed by optimizing crossflow velocities and using woven mesh supports. The draw solution will need to be reconcentrated; thus, a subsequent step like vacuum membrane distillation will be needed for potable water generation and regeneration of the draw solution. Compared to reverse osmosis, the attainable permeate flux likely will be lower, and water quality will need to be assessed. Ultimately, a technoeconomic analysis will be important to understand the cost–benefit relationships of the proposed process.

## Data Availability

The data required to reproduce these findings are available by request to the corresponding author.

## Supplementary Materials

The following are available online at https://www.mdpi.com/article/10.3390/membranes12010061/s1, Figure S1: Diagram of the homebuilt apparatus used in the direct flow testing, Figure S2: Piping and schematic of apparatus used in crossflow experimentation, Figure S3: Spacers used for mechanical support in the crossflow experimentation, Figure S4: Images of foulant on the feed side after testing in the direct flow system, Figure S5: Pictures of the membrane sides after experimentation in the crossflow system, Figure S6: Crossflow temperature measurements, Figure S7: Crossflow pressure measurements, Figure S8: Long-term crossflow flux measurements, Figure S9: Linear and Flory–Huggins model fits to experimental osmotic pressure data.
