# Supplementary material for: Water Recovery from Bioreactor Mixed Liquors Using Forward Osmosis with Polyelectrolyte Draw Solutions
Source: Membranes (Basel). 2021 Dec 31;12(1):61. doi: 10.3390/membranes12010061 (PMC8779258; doi:10.3390/membranes12010061)
Supplement: Supplementary file 1 [file membranes-12-00061-s001.zip › membranes-1520685-supplementary.pdf]

Supporting Information

Water recovery from bioreactor mixed liquors using forward osmosis with polyelectrolyte draw solutions

Calen R. Raulerson[a], Sudeep C. Popat[b], Scott M. Husson[a, *]

[a]Department of Chemical and Biomolecular Engineering, Clemson University, 127 Earle Hall, Clemson, SC 29634 USA

[b]Department of Environmental Engineering and Earth Sciences, Clemson University, 342 Computer Court, Anderson, SC 29625 USA

*Corresponding author

E-mail address: shusson@clemson.edu (S.M. Husson)

Contents

Figure S1. Diagram of the homebuilt apparatus used in the direct flow testing.

Figure S2. Piping and schematic of apparatus used in crossflow experimentation.

Figure S3. Spacers used for mechanical support in the crossflow experimentation.

Figure S4. Images of foulant on the feed side after testing in the direct flow system.

Figure S5. Pictures of the membrane sides after experimentation in the crossflow system.

Figure S6. Crossflow temperature measurements.

Figure S7. Crossflow pressure measurements.

Figure S8. Long-term crossflow flux measurements.

Figure S9. Linear and Flory-Huggins model fits to experimental osmotic pressure data.

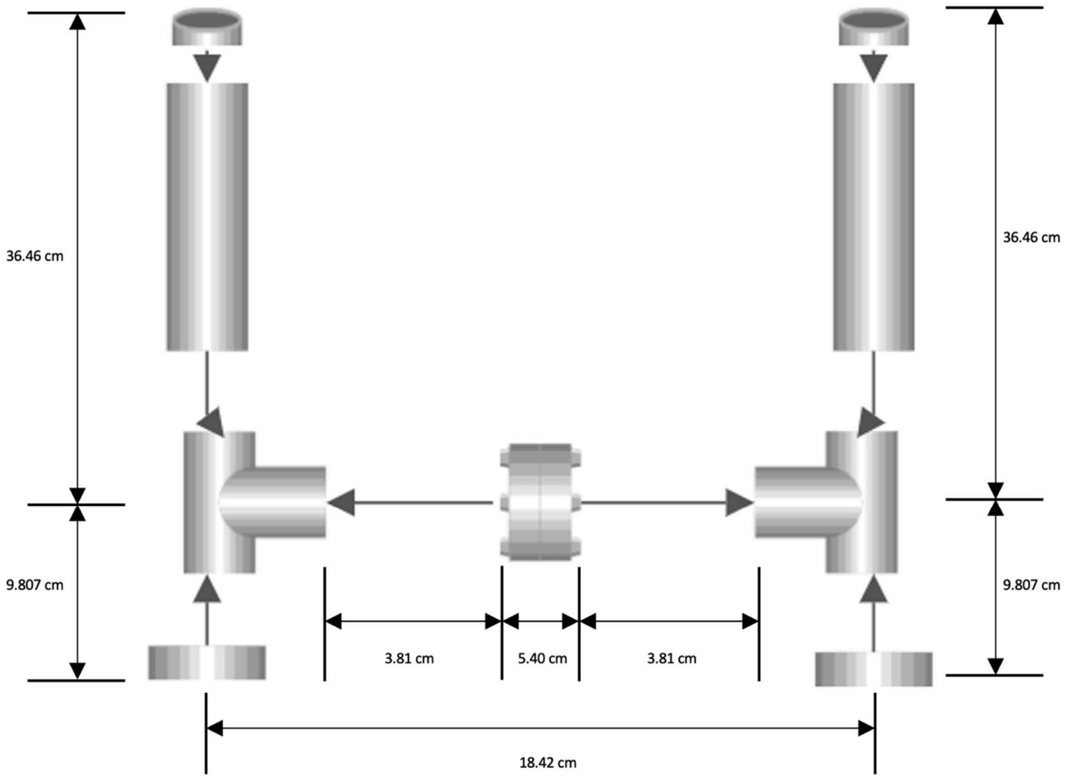

Figure S1. Diagram of the homebuilt apparatus used in the direct flow testing.

The materials were purchased from Grainger (Lake Forest, IL, USA) and consisted of 1’’ clear PVC pipe, 1’’ PVC flange fittings, 1’’ PVC Tee-bars, 1’’ PVC hex caps, and 1’’ rubber gaskets. All plastic components were glued together with PVC primer and PVC cement constructing an osmotic pressure device.

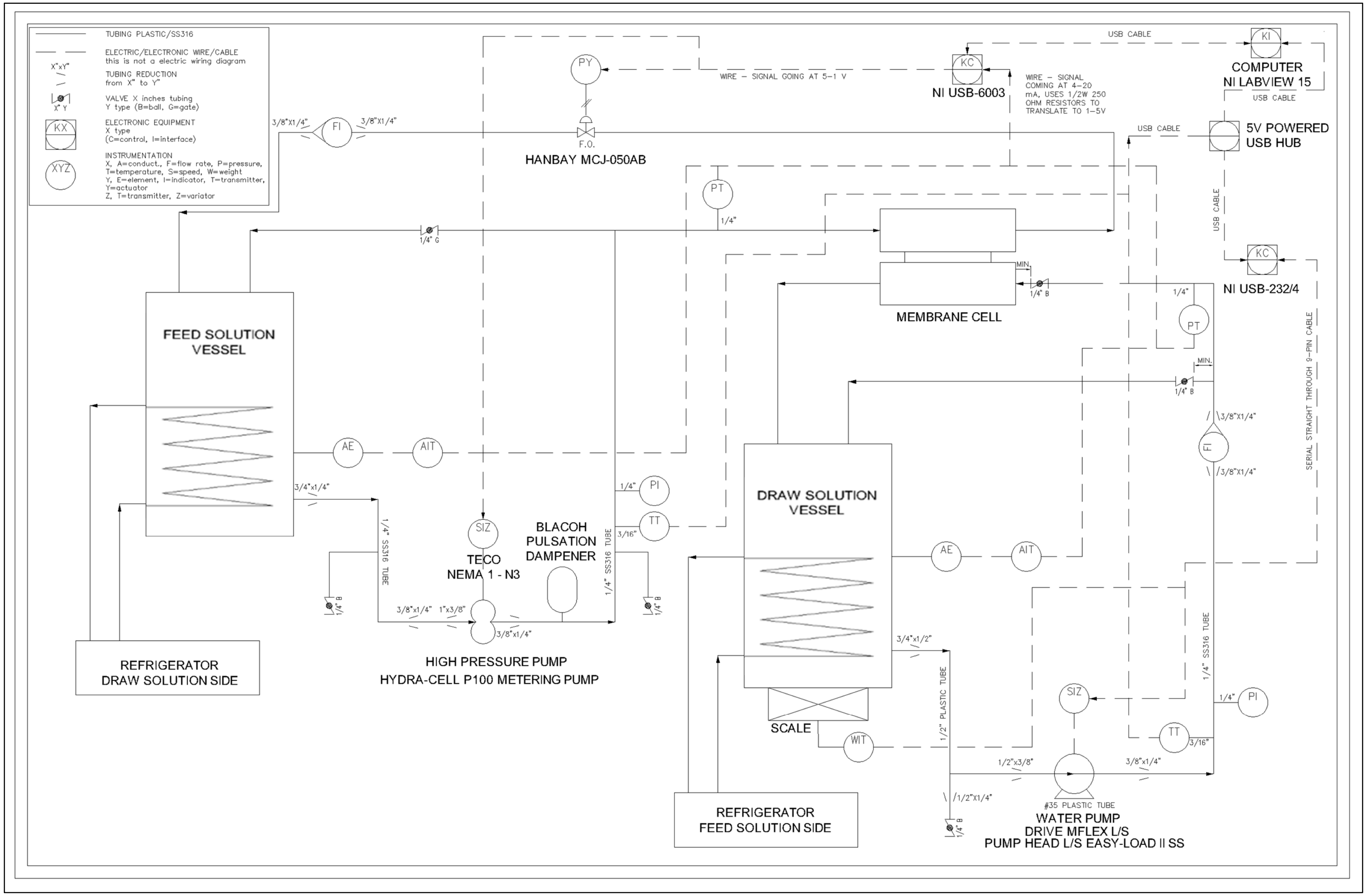

Figure S2. Piping and schematic of apparatus used in crossflow experimentation, adapted from Idarraga-Mora [7]. The feed and draw solutions have been swapped from the original work.

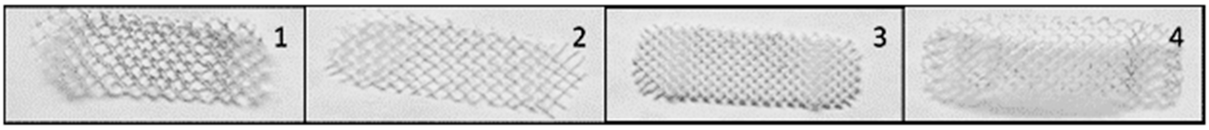

Figure S3. Spacers used for mechanical support in the crossflow experimentation.

The spacers were purchased from Spacers were purchased from Sterlitech Corporation (Kent, WA, USA). The spacers were used in order in their numbering: 1 was placed in closest contact to the membrane and 4 the furthest. These spacers applied the pattern seen in **Figure S5.**

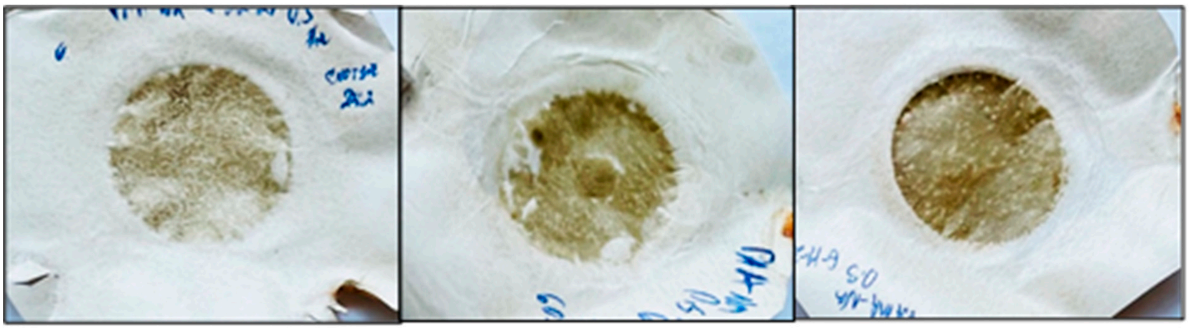

Figure S4. Images of fouling layer on the porous membrane side after testing in the direct flow system. The membrane is SW30HRLE.

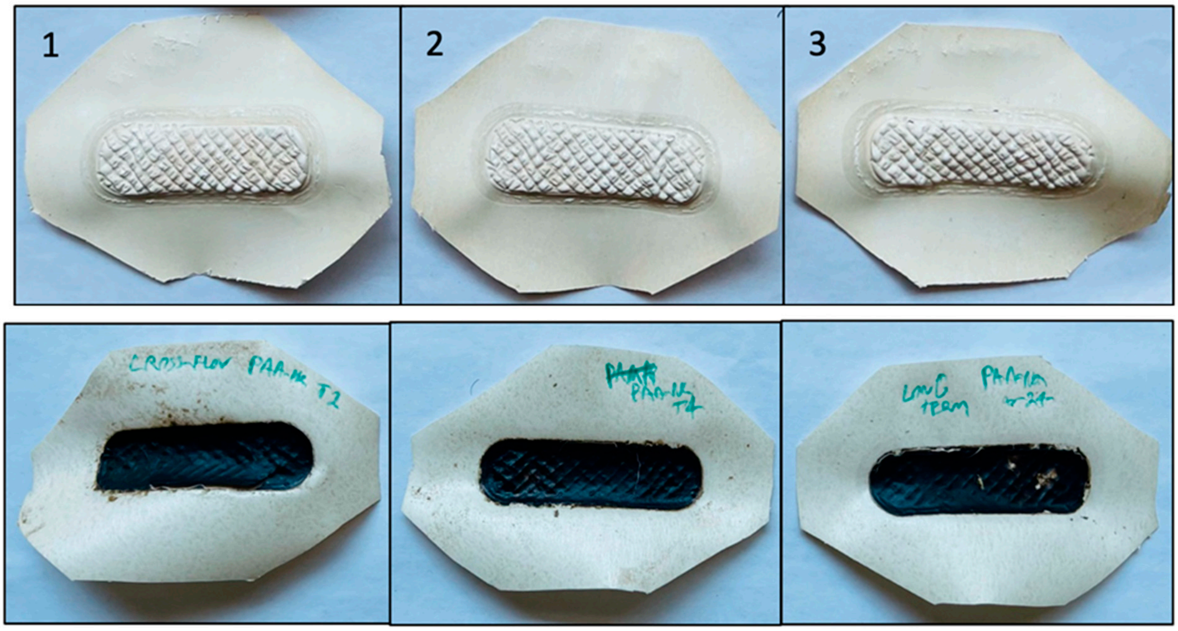

Figure S5. Pictures of the membrane sides after experimentation in the crossflow system. (Top) Draw solution side. (Bottom) Feed solution side.

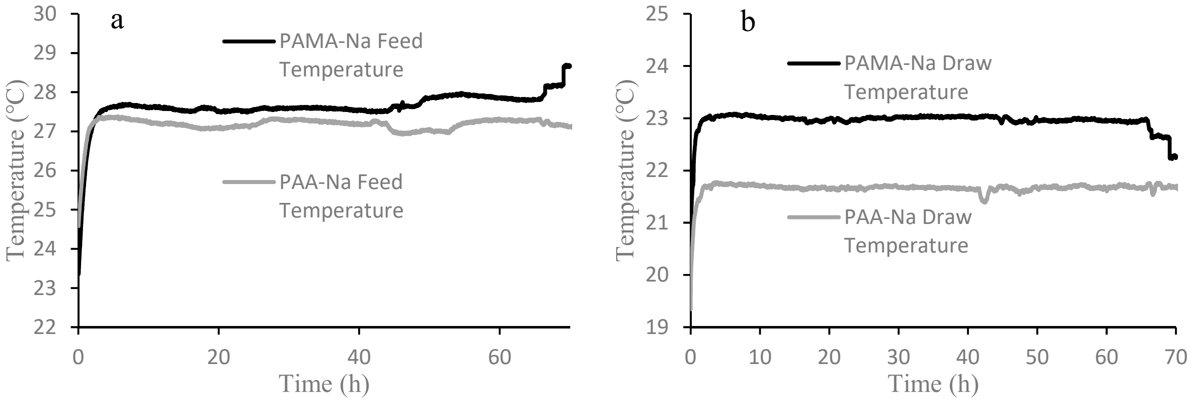

Figure S6. Crossflow temperature measurements for (a) feed solutions for PAA-Na and PAMA-Na trials at 0.3 g/mL and (b) draw solutions of PAA-Na and PAMA-Na at 0.3 g/mL.

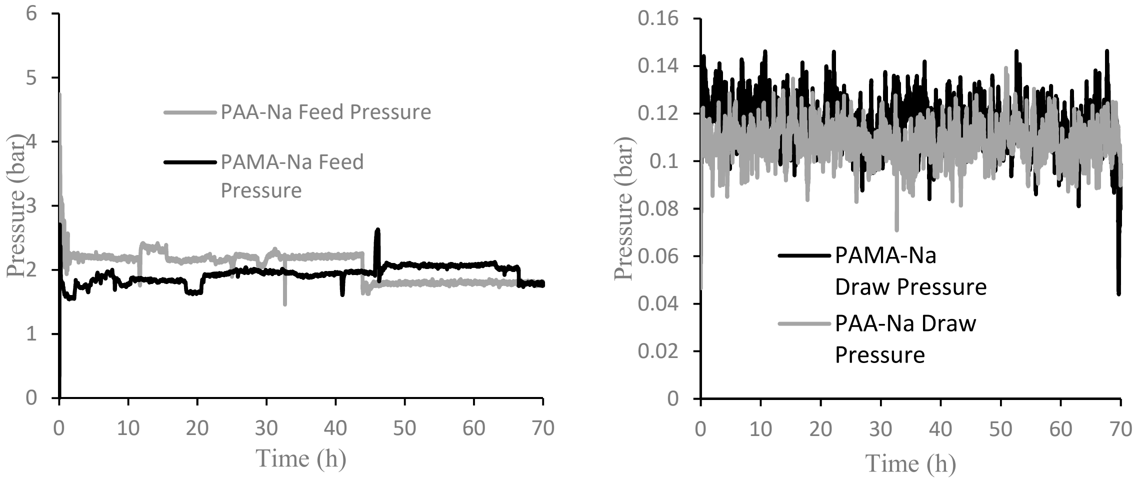

(a) (b)

Figure S7. Crossflow pressure measurements for (a) feed solutions for PAA-Na and PAMA-Na at 0.3 g/mL trials and (b) draw solutions of PAA-Na and PAMA-Na at 0.3 g/mL.

The temperature was maintained between 27 and 29 °C for optimal bioreactor conditions.

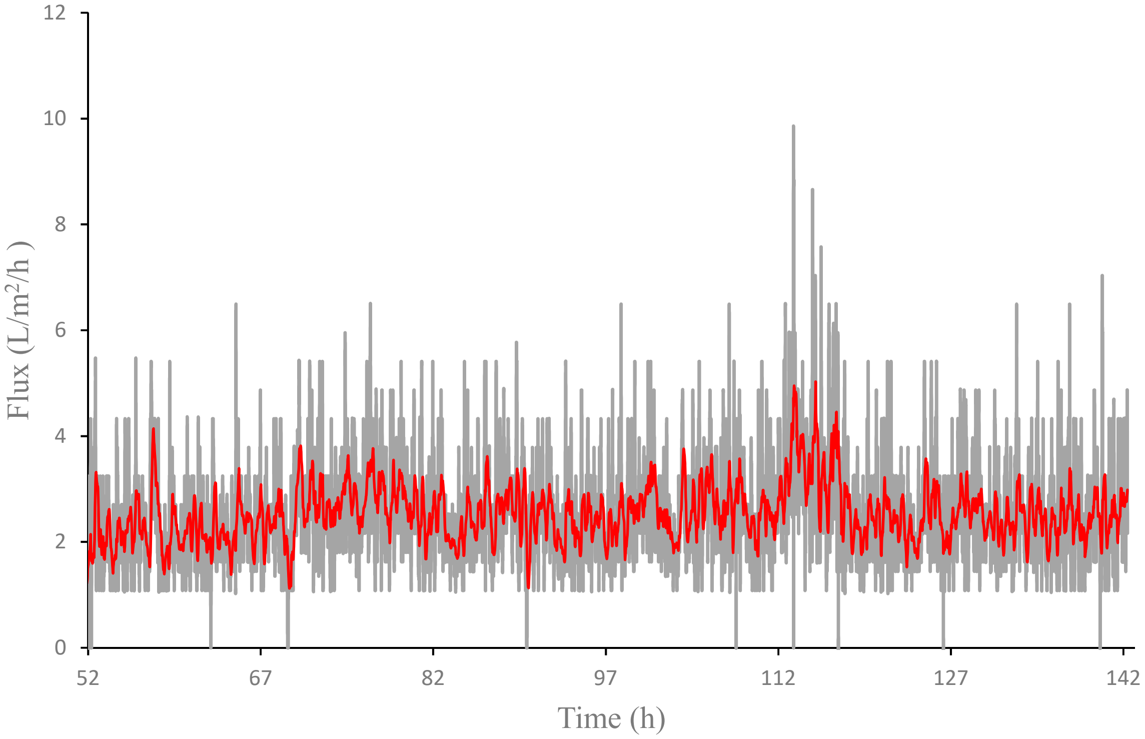

Figure S8. Crossflow flux measurements for PAA-Na at 0.3 g/mL extended to 143 h. The gray lines represent the raw data generated from three separate experiments and the red line represents the 20-point moving average.

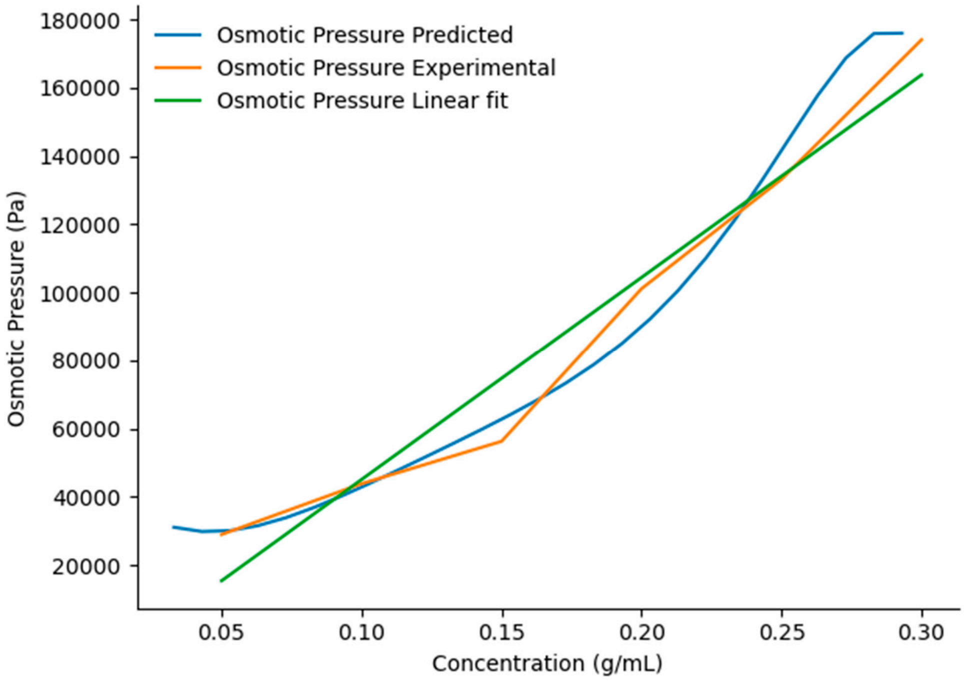

Figure S9. Linear and Flory-Huggins model fits to experimental osmotic pressure data. The Flory-Huggins model (blue curve) predicts a maximum in osmotic pressure around 0.28 g/mL PAA-Na concentration.
